# Linkage Rate Between Data From Health Checks and Health Insurance Claims in the Japan National Database

**DOI:** 10.2188/jea.JE20130075

**Published:** 2014-01-05

**Authors:** Etsuji Okamoto

**Affiliations:** National Institute of Public Health, Wako, Saitama, Japan; 国立保健医療科学院

**Keywords:** national database, record linkage, encryption, hash functions, health insurance claims

## Abstract

**Background:**

Japan’s National Database (NDB) includes data on health checks and health insurance claims, is linkable using hash functions, and is available for research use. However, the linkage rate between health check and health insurance claims data has not been investigated.

**Methods:**

Linkage rate was evaluated by comparing observed medical and pharmaceutical charges among health check recipients in fiscal year (FY) 2009 (*N* = 21 588 883) with expected charges from the same population when record linkage was complete. Using the NDB, observed charges were estimated from the first published result of linking health check recipients in FY2009 and their health insurance claims in FY2010. Expected charges were estimated by combining 3 publicly available datasets, including data from the Medical Care Benefit Survey and an ad-hoc report by the Japan Health Insurance Association.

**Results:**

Only 14.9% of expected charges were linked by the NDB. The linkage rate was higher for women than for men (18.2% vs 12.4%) and for elderly adults as compared with younger adults (>25% vs <10%).

**Conclusions:**

The linkage rate in the NDB was so low that any research linking health check and health insurance claims will not be reliable. Causes for the low linkage rate include differences between health check and health insurance claims data in name format (eg, insertion of a space between family and given names) and date of birth (Japanese vs Gregorian calendar). Investigation of the causes for the low linkage rate and measures for improvement are urgently needed.

## Abstract (ja)

**【背景】:**

日本のナショナルデータベース(NDB)はレセプトと特定健診を含んでおりハッシュ関数を用いた暗号によって同一人をリンケージ可能となっている。NDBは研究利用も認められているが,レセプトと特定健診がどれだけリンケージされているかは十分には評価されていない。

**【方法】:**

2009年度の特定健診受診者(N=21,588,883)の2010年度の医科調剤レセプトの医療費の推計額と, 実際にリンケージされた医療費の額とを比較した。実際にNDBでリンケージされた医療費の額は,厚生労働省が公表した2009年度特定健診受診者の性・年齢階級別の2010年度の医療費額を用いた。推計額は3つの公表データ(特定健康診査・特定保健指導の実施状況,医療給付実態調査そして全国健康保険協会による健診受診者・非受診者の分析)を合成して算出した。

**【結果】:**

NDBでリンケージされたレセプトの医療費額は,リンケージ率100%であれば予想される額のわずか14.9%にすぎなかった。リンケージ率は性・年齢階級別に異なっており,女性のリンケージ率は男性より高く(18.2% vs. 12.4%),また65歳以上は65歳未満よりもリンケージ率は高かった(65歳以上は25%以上であったのに対して65歳未満は10%未満)。

**【結論】:**

NDBにおけるレセプトと特定健診のリンケージ率はきわめて低く,両者を突合した研究結果は信頼性の面で疑問が残る。低いリンケージ率の原因としては,氏名の様式(レセプトでは姓名間にスペースを挿入する),生年月日の暦の違い(レセプトでは和暦,特定健診では西暦)等が考えられるが,正確な原因は不明である。すみやかにリンケージ率の低い原因(なぜ性・年齢階級で差があるのかも含め)を究明し,リンケージ率を向上させる対策をとることがNDBを用いた研究結果の信頼性を高めるためにも必要である。

## INTRODUCTION

In 2008, the National Database (NDB) was created in Japan for the “development, implementation, and evaluation” of the Health Care Cost Containment Plan (HCCCP), as set forth by Section 16 of the Elderly Health Care Security Act. Data from regular health checks and guidance have been collected since fiscal year (FY) 2008, and health insurance claims data have been collected since April 2009. The NDB has grown to one of the largest databases in the world and in June 2012 encompassed approximately 5 billion health insurance claims and 66 million health check and guidance data.^1^

Personally identifiable data in the NDB are irreversibly encrypted using hash functions. Because Japan does not have unique personal identifiers, 2 32-digit hash functions are generated: one from the insurer ID, beneficiary ID, date of birth, and sex, and the other from name, date of birth, and sex. By combining 2 hash functions, the NDB maximizes record linkage of health insurance claims with health check data from the same person.^2^

Unfortunately, the use of dual hash functions is by no means complete. Mistyping of names, inclusion of a space between family and first names, and a change in insurer or beneficiary ID will result in the generation of completely different hash functions, thereby compromising the accuracy of record linkage. Indeed, the accuracy of such record linkage in the NDB has not been fully investigated. The NDB is available for research use and many research projects using the NDB are underway.^3^ However, as a prerequisite of scientifically sound analysis, researchers must first ensure the accuracy of record linkage.

The author evaluated the linkage rate between health check data and health insurance claims in the NDB by comparing the medical and pharmaceutical charges observed for health check recipients in FY2009, ascertained through record linkage in the NDB, with the expected charges for the same population, estimated using publicly available data. If record linkage is complete, the observed and expected charges should match or at least be similar.

## METHODS

### Data source

Four publicly available datasets were used, all of which are available on the internet. The first 3 were used to estimate expected charges and the last one was used to estimate observed charges.

#### [1] Report on Health Checks and Guidance Regarding Metabolic Syndrome in FY2009^4^

The FY2009 Report on Health Checks and Guidance Regarding Metabolic Syndrome compiled administrative reports from 3453 insurers. It lists the number of beneficiaries “eligible for health checks”, which is defined as “beneficiaries as of April 1, 2009” and excludes those who quit in the middle of the fiscal year. For evaluation of insurer performance, the number of beneficiaries eligible for health checks is used as the denominator to calculate the percentage of health check recipients. Because insurers are held responsible only for beneficiaries eligible throughout the fiscal year, those who changed health insurance in the middle of the fiscal year are excluded from the denominator. However, in this study, the population as of October 1, 2009 was used as the denominator because the present study does not seek to evaluate insurer performance. Hence, the percentages of health check recipients reported in this study (males: 42.0%, females: 32.6%) are lower than those in the report (males: 46.5%, females: 36.4%) (Table 1).

**Table 1.** Percentages of individuals receiving health checks in fiscal year 2009

Age	MALES				FEMALES			
	
	Population as of Oct 2009 (in thousands, A)	Eligible for health check	Recipients of health check N(+)	% receiving health check, R N(+)/A	Population as of Oct 2009 (in thousands, A)	Eligible for health check	Recipients of health check N(+)	% receiving health check, R N(+)/A
40–44	4323	4 056 351	2 208 376	51.1%	4258	3 851 465	1 380 709	32.4%
45–49	3932	3 685 567	2 054 324	52.2%	3894	3 552 116	1 315 061	33.8%
50–54	3863	3 542 461	1 903 919	49.3%	3877	3 473 490	1 293 219	33.4%
55–59	4517	4 011 840	1 975 968	43.7%	4616	3 989 531	1 419 481	30.8%
60–64	4603	4 078 432	1 565 725	34.0%	4810	4 375 575	1 486 006	30.9%
65–69	4005	3 484 940	1 214 405	30.3%	4380	3 922 897	1 481 643	33.8%
70–74	3199	2 840 267	1 019 997	31.9%	3712	3 346 803	1 270 050	34.2%

Total	28 442	25 699 858	11 942 714	42.0%	29 547	26 511 877	9 646 169	32.6%

Source: Report of health check and guidance against metabolic syndrome in fiscal year 2009.

Source: http://www.mhlw.go.jp/bunya/shakaihosho/iryouseido01/dl/info03_h21_03.pdf.

#### [2] Analysis of data on health checks and medical charges in FY2008^5^

The Japan Health Insurance Association (JHIA) linked health check data and health insurance claims for 11 705 320 beneficiaries aged 35 to 74 years (the total was 9 618 145 when limited to individuals aged 40–74 years) in FY2008 and compared per capita charges between health check recipients and nonrecipients by sex and 5-year age group (Table 2).

**Table 2.** Per capita charges for medical and pharmaceutical claims by recipients and nonrecipients of health checks (annual charges in yen)

Age	MALES			FEMALES		
	
	Recipients P(+)	Nonrecipients P(−)	Ratio, r P(−)/P(+)	Recipients P(+)	Nonrecipients P(−)	Ratio, r P(−)/P(+)
40–44	68 460	83 017	1.21	80 391	93 937	1.17
45–49	89 120	112 220	1.26	92 159	107 617	1.17
50–54	117 000	148 787	1.27	109 917	128 035	1.16
55–59	149 394	197 421	1.32	128 347	154 232	1.20
60–64	191 084	257 593	1.35	158 692	195 420	1.23
65–69	235 556	339 828	1.44	199 147	258 205	1.30
70–74	332 376	512 231	1.54	290 377	403 938	1.39

Total	129 273	188 335	1.46	114 226	147 770	1.29

Source: Japan Health Insurance Association: Analysis of data on health checks and medical charges in fiscal year 2008.

Source: http://www.kyoukaikenpo.or.jp/~/media/Files/honbu/cat740/2506/250611/250611003.xls.

#### [3] Medical Care Benefit Survey, FY2011^6^

The Medical Care Benefit Survey (MCBS) is a population survey of all health insurance claims submitted from May 2011 thru April 2012 and is conducted by the Japan Ministry of Health, Labour and Welfare (MHLW). The FY2011 rather than the FY2010 MCBS was used because the MCBS included sex-specific data for the first time in FY2011. Because there was no fee schedule revision between FY2010 and FY2011, the estimates of charges will not be biased. Unlike the national database, which covers only electronically submitted claims, the MCBS includes all claims, including those submitted on paper, and thus provides the best estimate of per capita charges for the entire insured population. Since the MCBS is a survey of health insurance, it does not cover claims under the Livelihood Assistance Act for the indigent population. The MCBS also does not include Seamen’s Insurance, because the insurer did not submit the relevant data. In addition, some health insurance societies and mutual aid associations did not submit data and were thus excluded from the numerator and denominator. The survey report included age-specific number of beneficiaries as the denominator but no sex-specific data were available. Therefore, age- and sex-specific numbers of beneficiaries were estimated by applying sex ratios for the population as of October 1, 2011 (Table 3).

**Table 3.** Age- and sex-specific per capita medical and pharmaceutical charges for the entire population in fiscal year 2011

Age	MALES			FEMALES		
	
	No. of beneficiaries (N)	Medical and pharmaceutical charges in yen (C)	Per capita charges, P (C/N)	No. of Beneficiaries (N)	Medical and pharmaceutical charges in yen (C)	Per capita charges, P (C/N)
40–44	4 006 878	453 169 049 750	113 098	3 926 809	473 955 768 170	120 697
45–49	3 321 980	503 331 494 490	151 516	3 287 134	491 671 766 320	149 575
50–54	3 138 226	624 634 900 530	199 041	3 139 869	584 106 152 970	186 029
55–59	3 465 152	908 033 409 780	262 047	3 518 876	811 274 890 500	230 549
60–64	4 780 875	1 650 648 766 370	345 261	4 960 455	1 411 115 393 690	284 473
65–69	3 459 967	1 643 229 952 330	474 927	3 777 606	1 421 169 561 630	376 209
70–74	3 019 459	1 953 067 625 520	646 827	3 483 921	1 825 207 400 040	523 895

Total	25 192 537	7 736 115 198770	307 080	26 094 670	7 018 500 933320	268 963

Source: Medical Care Benefit Survey, fiscal year 2011

http://www.e-stat.go.jp/SG1/estat/GL02020101.do?method=xlsDownload&fileId=000006435815&releaseCount=1.

#### [4] Per capita medical and pharmaceutical charges for health check recipients in FY2009^7^

A report submitted by the MHLW to the Seventh Meeting of the Committee on Health Checks and Guidance on February 24, 2012 used hash functions to link health check data in FY2009 and health insurance claims data in FY2010 on an individual basis and was the first published evidence of the accuracy of record linkage in the NDB.

In FY2009, 21 588 883 beneficiaries (11 942 714 males and 9 646 169 females) underwent health checks. Of them, 2 685 509 beneficiaries (1 172 510 males and 1 512 999 females; 9.8% and 15.7%, respectively) were linked with FY2010 health insurance claims (medical, pharmaceutical, and diagnosis-procedure–combination [DPC]—a system of per diem payment for acute hospitals that is part of medical claims). The medical and pharmaceutical charges contained in the linked health insurance claims totaled 716 128 080 857 yen. Because the NDB contains only electronically submitted claims, the computerization rate of claims must be considered, to ensure fair comparison with the MCBS, which also contains claims submitted on paper. According to the Social Insurance Payment Fund, the computerization rate in FY2010 was 92.0% for medical claims and 99.9% for pharmaceutical claims, for an overall rate of 94.8%^8^ (463 225 000 medical and 281 613 000 pharmaceutical claims were submitted electronically out of 503 627 000 medical and 281 842 000 pharmaceutical claims in FY2010). The observed charges were inflated by multiplying values by the inverse of the computerization rate (Table 4).

**Table 4.** Observed medical and pharmaceutical charges for health check recipients linked to health insurance claims

Age	MALES			FEMALES		
	
	No. of health check recipients linked to health insurance claims n(+)	Per capita medical and pharmaceutical charges p(+)	Observed charges c(+) (n(+) * p(+))	No. of health check recipients linked to health insurance claims n(+)	Per capita medical and pharmaceutical charges p(+)	Observed charges c(+) (n(+) * p(+))
40–44	96 704	14 419	13 943 766 739	89 837	13 499	12 126 810 616
45–49	97 584	17 113	16 699 638 200	94 102	15 335	14 430 862 278
50–54	119 628	20 626	24 674 486 348	120 840	16 688	20 165 611 842
55–59	155 984	24 559	38 308 534 776	163 497	18 647	30 487 271 483
60–64	107 668	25 308	27 248 692 485	241 881	21 153	51 165 276 230
65–69	299 373	34 212	102 421 567 712	424 601	28 157	119 553 750 511
70–74	295 569	40 104	118 534 614 534	378 241	33 409	126 367 197 103

Total	1 172 510	29 154	3 418 313 00794	1 512 999	24 739	3 742 967 80063

Source: Per capita medical and pharmaceutical charges for health check recipients in fiscal year 2009.

Source: http://www.mhlw.go.jp/stf/shingi/2r98520000023mfn-att/2r98520000023mkh.pdf.

### Statistical analysis

Accuracy of the record linkage in the NDB was evaluated by comparing (1) the observed medical and pharmaceutical charges for health check recipients in data source [4] with (2) the expected medical and pharmaceutical charges of the same population estimated from data sources [1], [2], and [3]. It is expressed as c(+)/C(+) using the following notation:

N: number of beneficiaries obtained from data source [3]
N(+): number of health check recipients obtained from data source [1]
N(−): number of nonrecipients (= N − N(+))
n(+): number of health check recipients whose health insurance claims were linked using hash functions obtained from data source [4]
C: medical and pharmaceutical charges of all beneficiaries obtained from data source [3]
C(+): medical and pharmaceutical charges for health check recipients
C(−): medical and pharmaceutical charges for nonrecipients (= C − C(+))
c(+): medical and pharmaceutical charges for health check recipients whose health insurance claims were linked (ie, the observed medical and pharmaceutical charges for health check recipients)
P: per capita medical and pharmaceutical charges for all beneficiaries (= C/N)
P(+): per capita medical and pharmaceutical charges for health check recipients (= C(+)/N(+))
P(−): per capita medical and pharmaceutical charges for nonrecipients of health checks (= C(−)/N(−))
p(+): per capita medical and pharmaceutical charges for health check recipients whose health insurance claims were linked using hash functions obtained from data source [4] (= c(+)/n(+))

Subclassification was denoted by using the following subscripts:

i: sex (2 categories: males, females)
j: 5-year age group (7 categories: age 40–44, 45–49…70–74 years)
k: metabolic syndrome status (3 categories: no metabolic syndrome, risk of metabolic syndrome, metabolic syndrome)

#### Observed charges (c(+))

Observed medical and pharmaceutical charges for health check recipients, c(+), were calculated from data source [4], using the following formula (the results were inflated by the inverse of 0.948 to adjust for computerization of claims):

$$\sum_{i=1}^{2}\sum_{j=1}^{7}\sum_{k=1}^{3}n{\left(+\right)}_{ijk}*p{\left(+\right)}_{ijk}$$

#### Expected charges (C(+))

Expected medical and pharmaceutical charges, C(+), were estimated as:

C(+)=N(+)*P(+)

N(+) was obtained from data source [1]. P(+) had to be estimated from per capita charges for the entire population, obtained from data source [3]. Because bedridden people and hospitalized patients cannot receive health checks, the per capita charges for health check recipients (P(+)) should be lower than those for nonrecipients (P(−)).

Let r denote the ratio between per capita charges for nonrecipients over recipients, which was obtained from data source [2]:

r=P(−)/P(+)

Let R denote the percentage of those receiving health checks (= N(+)/N), as indicated in data source [1]. Then,

P(+)*N*R+P(−)*N*(1−R)=C

N*(P(+)*R+P(−)*(1−R))=N*P

Hence,

P(+)*R+P(−)*(1−R)=P

Since P(−) = r * P(+)

P(+)*R+r*P(+)*(1−R)=P

P(+)*(R+r−r*R)=P

∴P(+)=P/(R+r−r*R)

Using this formula, the per capita charges for health check recipients (P(+)) can be estimated. Then, C(+) is obtained as follows:

$$\sum_{i=1}^{2}\sum_{j=1}^{7}N{\left(+\right)}_{ij}*P{\left(+\right)}_{ij}$$

## RESULTS

The results are summarized in Table 5 and Figure.

**Figure.**
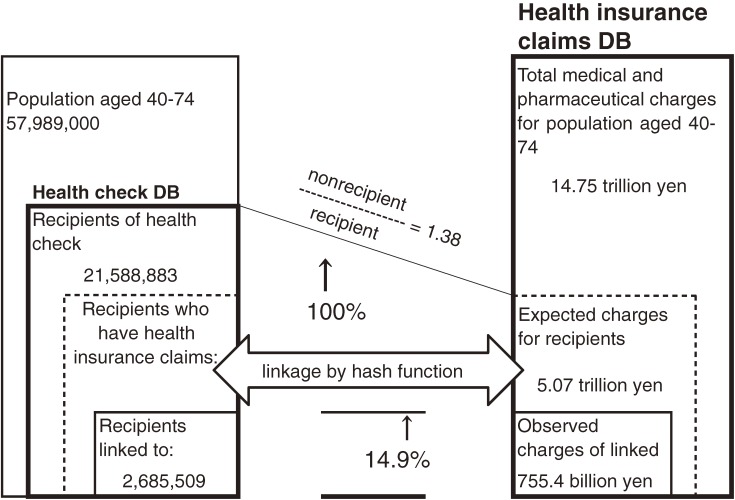
Summary of results.

**Table 5.** Observed and expected medical and pharmaceutical charges for health check recipients

Age	No. of health check recipients (N)	% receiving health check (R)	Ratio of charges of nonrecipients to recipients (r)	Per capita charges for entire population (in yen) (P)	Per capita charges for health check recipients (in yen) (P/(R + r − Rr))	Expected charges for health check recipients (N * P/(R + r − Rr))	Observed charges for health check recipients inflated by computerization rate (c(+)/0.948)	Observed/ Expected
MALES								
40–44	2 208 376	51.1%	1.21	113 098	102 443	226 231 655 926	14 708 614 704	6.5%
45–49	2 054 324	52.2%	1.26	151 516	134 827	276 978 115 882	17 615 652 110	6.4%
50–54	1 903 919	49.3%	1.27	199 041	174 938	333 067 001 956	26 027 939 186	7.8%
55–59	1 975 968	43.7%	1.32	262 047	221 915	438 495 975 016	40 409 846 810	9.2%
60–64	1 565 725	34.0%	1.35	345 261	280 776	439 617 687 810	28 743 346 503	6.5%
65–69	1 214 405	30.3%	1.44	474 927	362 972	440 795 159 536	108 039 628 388	24.5%
70–74	1 019 997	31.9%	1.54	646 827	472 625	482 076 124 377	125 036 513 222	25.9%
Subtotal	11 942 714	42.0%	1.46	307 080	242 744	2 899 018 186 819	360 581 540 922	12.4%
FEMALES								
40–44	1 380 709	32.4%	1.17	120 697	108 359	149 612 721 709	12 791 994 321	8.6%
45–49	1 315 061	33.8%	1.17	149 575	134 620	177 033 700 183	15 222 428 563	8.6%
50–54	1 293 219	33.4%	1.16	186 029	167 616	216 764 167 320	21 271 742 449	9.8%
55–59	1 419 481	30.8%	1.20	230 549	202 297	287 156 175 368	32 159 569 075	11.2%
60–64	1 486 006	30.9%	1.23	284 473	245 248	364 439 955 855	53 971 810 369	14.8%
65–69	1 481 643	33.8%	1.30	376 209	314 494	465 967 106 619	126 111 551 172	27.1%
70–74	1 270 050	34.2%	1.39	523 895	416 691	529 218 233 237	133 298 731 121	25.2%
Subtotal	9 646 169	32.6%	1.29	268 963	224 549	2 166 033 679 871	394 827 827 071	18.2%

Total	21 588 883	37.2%	1.38	287 686	232 281	5 065 051 866 690	755 409 367 993	14.9%

The NDB linked only 0.755 trillion yen of a total of 5.065 trillion yen actually charged for health check recipients in FY2009. Thus, in terms of charges, the NDB was able to link only 14.9% of health insurance claims.

There was an obvious sex difference: the linkage rate was higher for women than for men (18.2% vs 12.4%, respectively). In addition, there was an age difference: the linkage rate was higher for elderly adults than for younger adults. Adults aged 65 years or older had greater than 25% of their claims linked, while younger adults had less than 10% of their claims linked.

## DISCUSSION

The present results were alarming. The linkage rate of 14.9% was far lower than that of the Japan Medical Data Center (JMDC) database (88.5% with 1 hash function and 98.0% with 2 hash functions combined)^9^ and might bias the findings of any research linking health check and health insurance claims data. The NDB was created for the “development, implementation and evaluation” of the HCCCP, which emphasizes health care cost containment through prevention of metabolic syndrome. However, the low linkage rate of the NDB makes it incapable of fulfilling that task.

The reasons for the low linkage rate and sex and age differences are not clear. One possibility is that the formats for names and dates of birth are inconsistent on the health insurance claims and health check data. A space must be inserted between family and given names on health insurance claims but not in health check data. Although date of birth is recorded using the Japanese calendar for health insurance claims, it is recorded using the Gregorian calendar for health checks.

The advantage of this study is that it is based entirely on publicly available datasets, thanks to the recent availability of detailed data. One such development is the availability of per capita charges for health check recipients and nonrecipients from the Japan Health Insurance Association. The fact that male nonrecipients of health checks consume 1.46 times the charges of recipients sheds new light on the conventional wisdom that municipalities with higher health check participation have lower per capita health care charges. Another development was the Medical Care Benefit Survey, which serves as a “mirror site” of the NDB. Interestingly, different sections of the MHLW collect the same health insurance claims data based on different legal requirements.^3^ These dual databases provided the author a valuable opportunity to obtain observed and expected charges by means of comparing them.

This study did have limitations, however. Although it revealed the low linkage rate of the NDB, the reasons for this low linkage remain unclear. Investigation of the low linkage rate and identification of measures for improvement are thus urgently needed. Hash function encryption is performed by the Prefectural Federations of National Health Insurance and by prefectural branches of the Social Insurance Payment Fund, using an encryption program distributed by the MHLW (not by individual health insurers). The author suspects that the encryption algorithm is flawed, although this would not fully explain the observed sex and age differences. Since hash functions are irreversible, it is not possible to investigate causes within the NDB.

A future field test involving health insurers of sufficient enrollment size may be useful. By comparing the original, personally identifiable data (health insurance claims and health check data) with the encrypted data generated by the encryption program, it would be possible to identify the causes for the low linkage rate. Once these causes are identified, the encryption algorithm should be revised, and the old data, back to April 2009, should be recollected before they are lost, as it is not too late to address the problem.

### Suggestions for researchers

The NDB is available for research use, and publications based on NDB data are already appearing. However, researchers and reviewers must carefully consider the linkage rate using hash functions, as it should never be assumed that the linkage is 100%. Just as response rate is required in reporting a questionnaire survey, linkage rate should be reported when using NDB data, particularly when using data to link the same individual across time or attempting to link health check and health insurance claims data.

This study provides a method for evaluating linkage rate. Researchers who use NDB data should refer to its mirror site, the MCBS. Because the MCBS covers the same health insurance claims as the NDB (actually the coverage of the MCBS is greater because it covers health insurance claims submitted on paper), researchers should be able to compare health care charges on a sex- and age-specific basis.

As a matter of policy, researchers are prohibited from cross-linking the NDB with any other individual-level data. However, this does not preclude comparisons with or references to other publicly available aggregate data. Researchers are reminded that part of the NDB is publicly available. The Social Insurance Claims Survey has collected data directly from the NDB for hospitals and pharmacies since 2011. Pharmacy MEDIAS collects electronic pharmacy claims since 2004 but was replaced by the NDB in April 2012.^10^ Finally, the JHIA independently provides aggregate data on health insurance claims. By comparing those publicly available aggregate data, researchers may be able to evaluate linkage rate and any potential bias related to it.

## ONLINE ONLY MATERIALS

Abstract in Japanese.
