# Supplementary material for: Linkage Rate Between Data From Health Checks and Health Insurance Claims in the Japan National Database
Source: J Epidemiol. 2014 Jan 5;24(1):77–83. doi: 10.2188/jea.JE20130075 (PMC3872528; doi:10.2188/jea.JE20130075)
Supplement: Abstract in Japanese. [file je-24-077-s001.pdf]

タイトル:ナショナルデータベースにおけるレセプトと特定健診データのリンケージ率

岡本悦司
国立保健医療科学院

【背景】日本のナショナルデータベース(NDB)はレセプトと特定健診を含んでおりハッシュ関数を用いた暗号によって同一人をリンケージ可能となっている。NDB は研究利用も認められているが,レセプトと特定健診がどれだけリンケージされているかは十分には評価されていない。

【方法】2009 年度の特定健診受診者(N=21,588,883)の 2010 年度の医科調剤レセプトの医療費の推計額と, 実際にリンケージされた医療費の額とを比較した。実際に NDB でリンケージされた医療費の額は,厚生労働省が公表した 2009 年度特定健診受診者の性・年齢階級別の 2010 年度の医療費額を用いた。推計額は 3 つの公表データ(特定健康診査・特定保健指導の実施状況,医療給付実態調査そして全国健康保険協会による健診受診者・非受診者の分析)を合成して算出した。

【結果】NDB でリンケージされたレセプトの医療費額は,リンケージ率 100%であれば予想される額のわずか 14.9%にすぎなかった。リンケージ率は性・年齢階級別に異なっており,女性のリンケージ率は男性より高く(18.2% vs. 12.4%),また 65 歳以上は 65 歳未満よりもリンケージ率は高かった(65 歳以上は 25%以上であったのに対して 65 歳未満は 10%未満)。

【結論】NDB におけるレセプトと特定健診のリンケージ率はきわめて低く,両者を突合した研究結果は信頼性の面で疑問が残る。低いリンケージ率の原因としては,氏名の様式(レセプトでは姓名間にスペースを挿入する),生年月日の暦の違い(レセプトでは和暦,特定健診では西暦)等が考えられるが,正確な原因は不明である。すみやかにリンケージ率の低い原因(なぜ性・年齢階級で差があるのかも含め)を究明し,リンケージ率を向上させる対策をとることが NDB を用いた研究結果の信頼性を高めるためにも必要である。

キーワード:ナショナルデータベース,レコードリンケージ,暗号化,ハッシュ関数,レセプト
